# Comparative transcriptomic analysis reveals differences in gene expression and regulatory pathways between nonacral and acral melanoma in Asian individuals

**DOI:** 10.1111/1346-8138.17187

**Published:** 2024-03-12

**Authors:** Yu‐Jen Chiu, Cheng‐Yuan Li, Tien‐Hsiang Wang, Hsu Ma, Teh‐Ying Chou

**Affiliations:** ^1^ Institute of Clinical Medicine National Yang Ming Chiao Tung University Taipei Taiwan; ^2^ Division of Plastic and Reconstructive Surgery, Department of Surgery Taipei Veterans General Hospital Taipei Taiwan; ^3^ Department of Surgery, School of Medicine National Yang Ming Chiao Tung University Taipei Taiwan; ^4^ Department of Dermatology Taipei Veterans General Hospital Taipei Taiwan; ^5^ Institute of Brain Science National Yang Ming Chiao Tung University Taipei Taiwan; ^6^ Department of Surgery, School of Medicine National Defense Medical Center Taipei Taiwan; ^7^ Department of Pathology and Laboratory Medicine Taipei Veterans General Hospital Taipei Taiwan; ^8^ Department of Pathology and Precision Medicine Research Center Taipei Medical University Hospital Taipei Taiwan; ^9^ Graduate Institute of Clinical Medicine, College of Medicine Taipei Medical University Taipei Taiwan

**Keywords:** Asians, melanoma, RNA sequencing, transcriptome

## Abstract

Melanoma predominantly occurs in White individuals, which is associated with factors such as exposure to UV radiation and skin pigmentation. Despite its low incidence, melanoma is the primary cause of skin cancer–related death in Asia, typically in areas with low sun exposure. In our previous whole‐exome sequencing study, we identified mutational signature 12 as the most prevalent variant in Asian patients, differing from the common UV‐associated mutational signature 7 observed in White individuals. We also observed major differences between acral melanoma (AM) and nonacral melanoma (NAM) in terms of signatures 7, 21, and 22. Notably, few studies have investigated the genomic differences between AM and NAM in Asian individuals. Therefore, in this study, we conducted transcriptomic sequencing to examine the disparities in RNA expression between AM and NAM. Ribosomal RNA depletion was performed to enhance the detection of functionally relevant coding and noncoding transcripts. Ingenuity pathway analysis revealed significant differences in gene expression and regulatory pathways between AM and NAM. The results also indicate that the genes involved in cell cycle signaling or immune modulation and programmed cell death protein 1/programmed cell death 1 ligand 1 signaling were differentially expressed in NAM and AM. In addition, high CDK4 expression and cell cycle variability were observed in AM, with high immunogenicity in NAM. Overall, these findings provide further insights into the pathogenesis of melanoma and serve as a reference for future research on this major malignant disease.

## 1 INTRODUCTION

Melanoma is a malignant neoplasm that originates from melanocytes and represents a major health concern. Despite accounting for only a small proportion of all skin cancer cases, melanoma is responsible for the majority of skin cancer–related deaths.^1^ Recently, a global increase has been observed in the incidence of melanoma, particularly in Western countries, where UV exposure and fair skin types are associated with an increased risk of melanoma.^2^ Traditionally, melanoma is primarily treated using surgical resection, radiation therapy, and chemotherapy. However, recent advancements have led to the introduction of various treatment options, including targeted therapy and immunotherapy, and have revolutionized patient prognosis.^3^ These treatment options include BRAF and MEK inhibitors and immune checkpoint inhibitors, such as cytotoxic T lymphocyte–associated protein 4 and programmed cell death protein 1/programmed cell death 1 ligand 1 (PD‐1/PD‐L1) inhibitors. These treatment options are associated with major improvements in overall survival rates. Despite these advancements, the heterogeneous nature of melanoma often leads to poor therapeutic responses and the development of drug resistance, thereby indicating the need for ongoing research.^3^, ^4^, ^5^

Compared with Western countries, Asian countries, including Taiwan, report a lower incidence of melanoma.^6^ However, in Asian countries, melanoma is often diagnosed at more advanced stages, resulting in lower response rates to targeted therapy and immunotherapy, which, in turn, leads to a disproportionately high mortality rate.^7^, ^8^, ^9^, ^10^, ^11^ These unique challenges are influenced by racial and regional differences in the disease profile. To address these challenges, substantial research efforts have been made in the development of melanoma treatment in Asian populations. Specifically, Taiwanese researchers have comprehensively examined the genetic landscape of melanoma, aiming to uncover unique genetic signatures that can be targeted for therapy.^12^, ^13^, ^14^ In our previous study, which is regarded as the first whole‐exome sequencing (WES) study to be conducted in Asian individuals, we investigated the differences in the genomic profiles of melanoma between Asian and White individuals.^15^ We discovered that in Taiwanese patients, mutational signature 12 was the predominant variant, accounting for approximately 45% of the observed mutational signatures. By contrast, according to The Cancer Genome Atlas (TCGA), mutational signature 7 (UV mutational signature) is the predominant variant in White patients.^16^ In addition, our previous study revealed a significantly lower tumor mutation burden (TMB) in our cohort compared with that in White individuals.^15^ In Asian individuals with melanoma, reduced UV signature and TMB may contribute to a lower BRAF mutation rate and a lower response rate to immunotherapy.^17^, ^18^ Therefore, further research is required to address these challenges and improve the outcomes of melanoma in Asia.

According to previous genomic studies on melanoma in White individuals, each melanoma subtype has a unique signature landscape and unique mutations. For instance, nonacral melanoma (NAM) is primarily attributable to UV radiation and is associated with increased BRAF mutations.^19^ By contrast, acral melanoma (AM) is associated with lower UV signatures and mutations but exhibits additional structural changes.^20^ To the best of our knowledge, few studies have examined the genomic differences between the different subtypes of melanoma in Asian individuals. In our previous study, although we identified mutational signature 12 as the predominant variant, we observed significant differences between AM and NAM in terms of certain mutational signatures, such as in mutational signatures 7, 21, and 22.^15^ We also discovered that NAM samples exhibited significantly high concentrations of mutational signatures 7, 21, and 22 along with increased TMB and microsatellite instability (MSI).^15^ In this study, after the analysis of various DNA sequences, we used transcriptome sequencing to examine the differences in RNA expression between AM and NAM.

## 2 MATERIALS AND METHODS

### 2.1 Patients and specimens

This retrospective cohort study was approved by the institutional review board of Taipei Veterans General Hospital (approval numbers 2022‐06‐007 A, 2021‐05‐002 AC, and 2020‐01‐013 BC). The hospital's patient database was used to identify patients with melanoma who received their diagnoses from 2010 to 2021. After the diagnoses were confirmed, the percentages of tumor cells were estimated by pathologists. Subsequently, the National Comprehensive Cancer Network guidelines were employed to ensure clinicopathological staging. In accordance with hospital protocol, formalin‐fixed paraffin‐embedded (FFPE) block samples were collected from 23 primary cutaneous melanomas (15 AM and eight NAM samples) paired with normal tissues.

### 2.2 Nucleic acid extraction and ribosomal RNA depletion

A Covaris truXTRAC FFPE Total NA Plus Kit (category number 520252, Covaris) was used to isolate RNA from lysates as per the manufacturer's instructions. An Agilent 2100 Bioanalyzer (Agilent Technologies) equipped with an RNA nanochip was used to evaluate the quality and quantity of RNA. Agilent 2100 Expert Software was used to calculate DV200, a measure of RNA integrity that represents the percentage of fragments exceeding 200 nucleotides. A NanoDrop spectrophotometer (Thermo Fisher Scientific) was used to evaluate the quality of RNA. Ribosomal RNA (rRNA) depletion was performed for 0.5 μg of the total RNA per sample. rRNA was selectively removed using biotinylated, target‐specific oligonucleotides in conjunction with Ribo‐Zero rRNA removal beads. All subsequent steps were performed using a TruSeq Stranded Total RNA Library Prep Gold Kit (category number 20020598, Illumina) in accordance with the respective manuals.^21^, ^22^, ^23^

### 2.3 Whole‐transcriptome sequencing

Total RNA extraction and quantitation, sequencing cluster generation, and high‐throughput sequencing were performed in accordance with the protocols outlined by Illumina (NEB). An Illumina TruSeq Stranded Total RNA Library Prep Gold Kit (categary number 20020598) was used for library construction, followed by AMPure XP bead (Beckman Coulter) size selection. Sequences were identified using Illumina's sequencing‐by‐synthesis technology (Illumina). Sequencing data (FASTQ reads) were generated using Welgene Biotech's pipeline in Illumina's base‐calling program bcl2fastq version 2.20.

The data‐trimming process involved binary base call (BCL) conversion, adaptor clipping, and sequence quality trimming. The BCL files obtained from all Illumina sequencing platforms were converted into FASTQ reads in Illumina's bcl2fastq conversion software version 2.20. Short nucleotide sequences, which are also known as adaptors and are essential for next‐generation sequencing (NGS), were eliminated, and low‐quality reads or bases were trimmed, especially from the 3′ end, by using a sliding‐window approach in Trimmomatic version 0.36. NGS reads were aligned to genomes by using HISAT2, a rapid and sensitive program that relies on a hierarchical graph FM index for swift and precise alignment. In HISAT2, a small graph FM index set was used, which was particularly effective for splice junction alignment.

For differential gene discovery, the default settings were set as a cutoff *p* value of 0.05 and an abs (log2.0FC) cutoff of 1.00. Because of the potential overestimation of the false discovery rate without sufficient replicates, the *q*‐value, which measures the false discovery rate, was not set by default. StringTie (version 2.1.4) and DESeq (version 1.39.0) or DESeq2 (version 1.28.1) was used to conduct a differential expression analysis, and genome bias detection and correction were conducted in Welgene Biotech's in‐house pipeline. Functional enrichment analysis of differentially expressed genes was conducted using clusterProfiler version 3.6. Low‐expression genes (<0.3 TPM) were excluded, and genes with a *p* value of <0.05 and >2.0‐fold changes were considered to exhibit significant differential expression.^24^, ^25^

### 2.4 Ingenuity pathway analysis

Network analysis was conducted using ingenuity pathway analysis (IPA). Total target genes were organized as focus molecules and analyzed using a core analysis tool in IPA software (IPA 2021, QIAGEN Sciences). Fisher exact *t* tests were conducted to calculate enriched networks and associated ontology groups with their respective upstream regulators, canonical pathways, functions, diseases, and network analysis rankings on the basis of statistical significance (*p* < 0.05). The molecular networks in the QIAGEN knowledge base originated from the focus molecules, and each molecule was connected to others. This analysis was conducted in duplicate.^26^

### 2.5 Immunohistochemical staining

Immunohistochemistry was performed according to standard procedures. Briefly, the FFPE tissue sections were deparaffinized, subjected to antigen retrieval, and followed by incubation with primary antibodies specifically targeting CD8 (clone SQab19146, Arigo Biolaboratories), PD‐1 (clone SQab1732, Arigo Biolaboratories), and PD‐L1 (clone SQab1716, Arigo Biolaboratories). Subsequent to primary antibody application, sections were incubated with orseradish peroxidase–conjugated secondary antibodies, washed, and then developed into the desired staining intensity. Counterstaining was performed with hematoxylin and mounted with a permanent mounting medium.

### 2.6 Reverse transcription quantitative polymerase chain reaction analysis

RNA extracted from the FFPE samples of 12 AM and five NAM tumors were used to quantify the expression levels of CD8, PD‐1, and PD‐L1 by reverse transcription quantitative polymerase chain reaction (RT‐qPCR) analysis according to standard procedures. Briefly, following complementary DNA synthesis, qPCR was performed using specific primer sets designed for CD8, PD‐1, and PD‐L1, alongside a reference gene for normalization of expression data. One Step SYBR PrimeScript RT‐qPCR kit II (Takara Bio, Inc.) was applied for detection of the amplified product. Primers were synthesized by Mission Biotech. The primer sequences for qPCR are as follows: Human CD8‐F: 5′‐GGACTTCGCCTGTGATATCTACATC‐3′; Human CD8‐R: 5′‐GGACATTTGCAAACACGTCTTC‐3′; Human CD274‐F: 5′‐GTGCCGACTACAAGCGAATTACT‐3′; Human CD274‐R: 5′‐AGCCCTCAGCCTGACATGTC‐3′; Human PDCD1‐F: 5′‐GATGGTTCTTAGACTCCCCAGACA‐3′; Human PDCD1‐R: 5′‐GAAGGCGGCCAGCTTGT‐3′. The data were statistically analyzed by one‐way analysis of variance followed by Dunnett test using SPSS software version 28.0 (IBM). A *p*‐value <0.05 was considered to be statistically significant and is marked with an asterisk in the figures.

## 3 RESULTS

### 3.1 Comprehensive transcriptomic profiling reveals gene expression dynamics in NAM and AM

**FIGURE 1:**
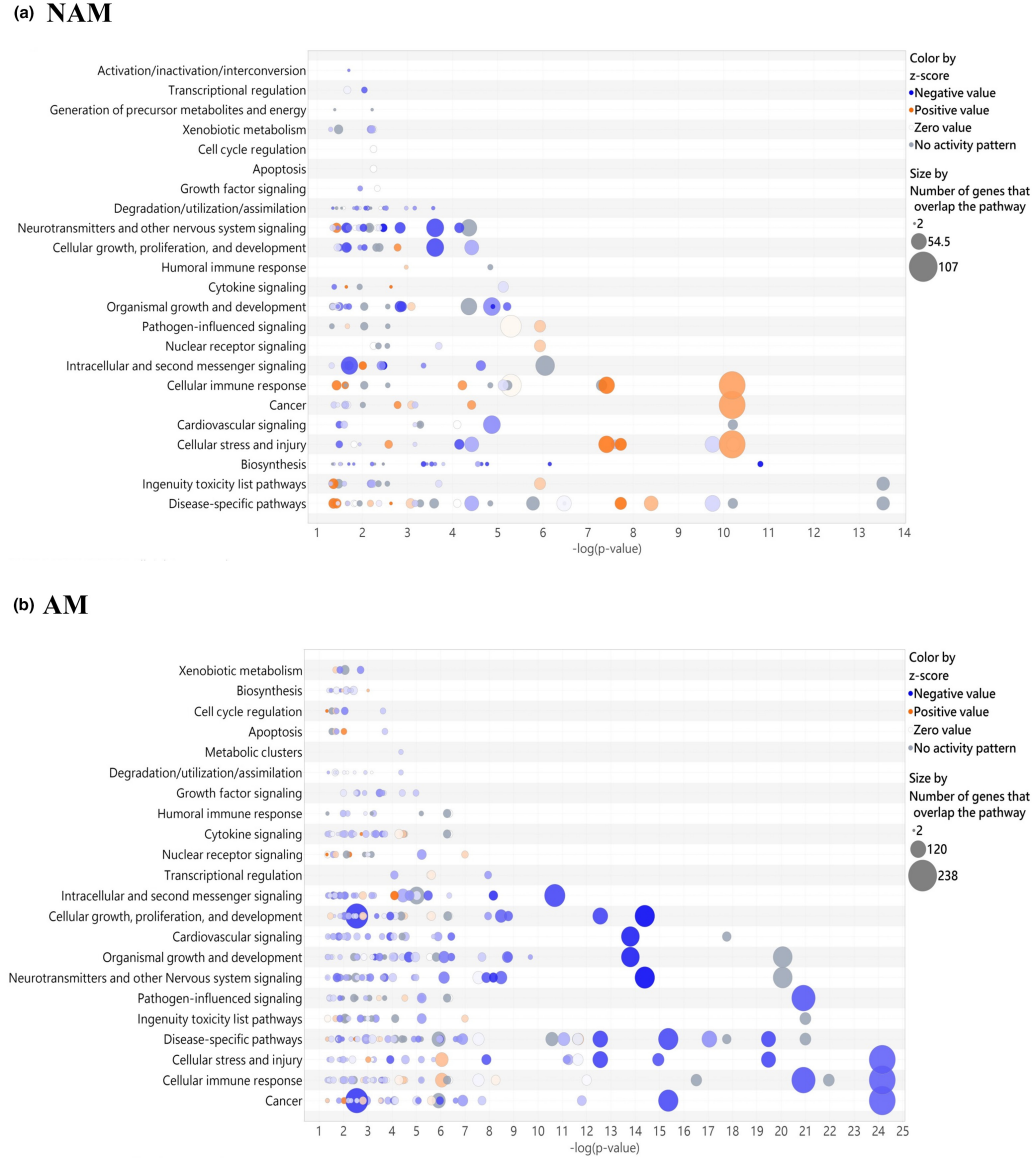
Bubble plots revealing the differences in gene count and regulatory pathways between nonacral melanoma (NAM) and acral melanoma (AM). The size of each bubble represents the number of genes overlapping with the pathways. Blue bubbles represent negative values, and orange bubbles represent positive values. (a) In the case of NAM, most of the upregulated genes were distributed in the cellular stress and injury pathway, cancer pathway, and cellular immune response pathway. (b) In the case of AM, most of the downregulated genes were distributed in the cellular stress and injury pathway, cancer pathway, and cellular immune response pathway.

**FIGURE 2:**
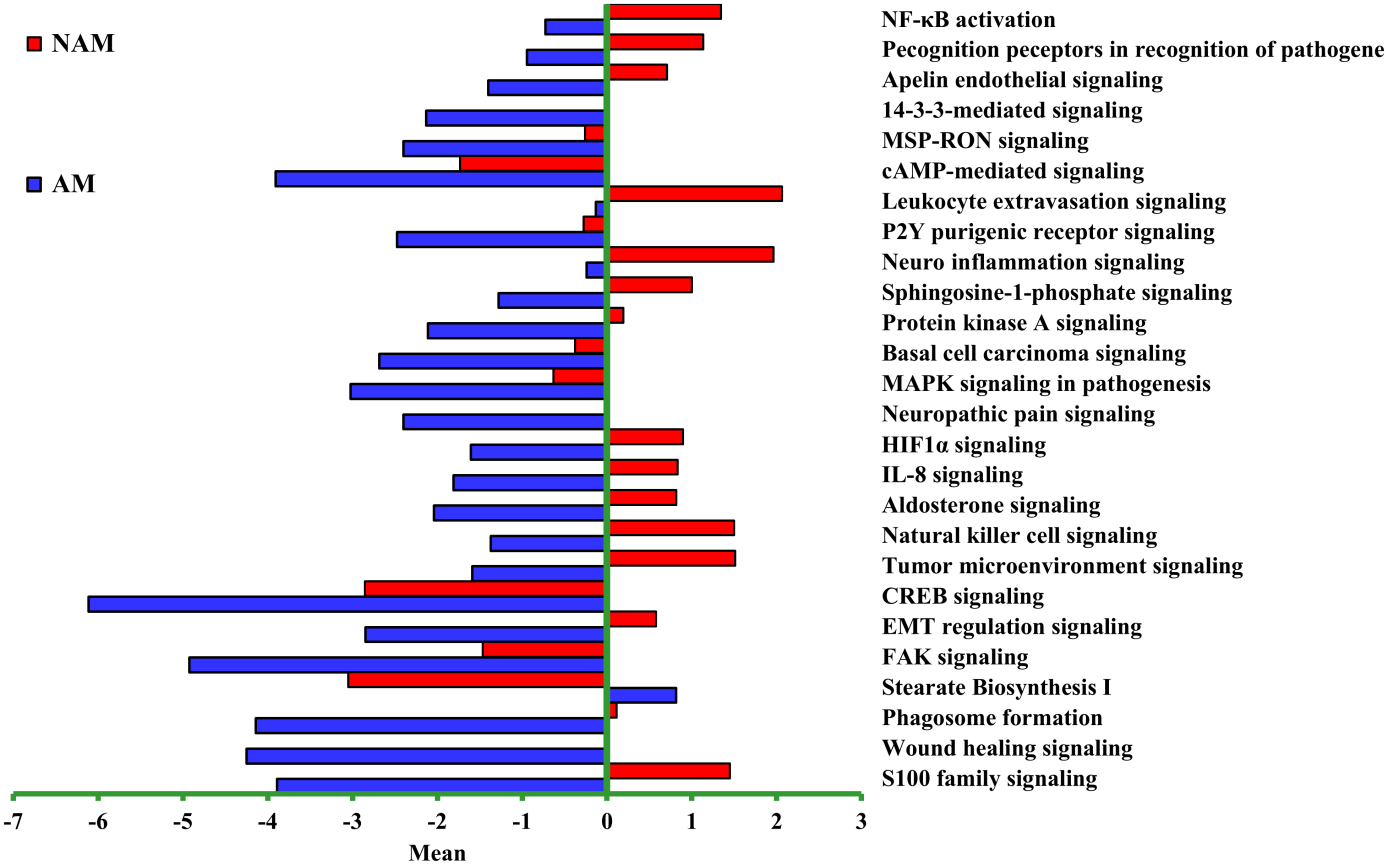
Differences in signaling pathways between nonacral melanoma (NAM) and acral melanoma (AM). Each horizontal bar denotes a distinct pathway with a unique *z* score. Red bars indicate activation, and blue bars indicate suppression. The following pathways were identified as the most significantly different regulatory pathways between NAM and AM: S100 protein family signaling pathway, wound healing signaling pathway, phagosome formation pathway, stearate biosynthesis I pathway, focal adhesion kinase (FAK) signaling pathway, epithelial mesenchymal transition (EMT) regulation signaling pathway, cAMP‐response element‐binding protein signaling pathway (CREB), tumor microenvironment signaling pathway, and natural killer cell signaling pathway. HIF1α, hypoxia‐inducible factor 1 α; IL‐8, interleukin 8; MAPK, mitogen‐activated protein kinase; NF‐κB, nuclear factor–κB.

According to TCGA and the Translational Genomics Research Institute melanoma data set, common gene mutations are not frequently detected in Taiwanese patients. In addition, major specific gene mutations occur in AM but not in NAM.^15^ In this study, to elucidate the differences in gene expression and signaling pathways between NAM and AM, RNA sequencing and IPA analysis were conducted on primary melanomas (15 AM and eight NAM samples) paired with normal tissues. Clinical characteristics of patients undergoing transcriptome sequencing are presented in Table 1. rRNA depletion was performed for enhanced detection of coding and noncoding transcripts. Bubble plots revealing the differences in gene count and regulatory pathways between NAM and AM are presented in Figure 1. The number of genes and regulatory pathways differed between NAM and AM. In NAM, most of the upregulated genes were distributed in the cellular stress and injury pathway, cancer pathway, and cellular immune response pathway (Figure 1a, orange points). In AM, most of the downregulated genes were distributed in the intracellular and second messenger signaling pathway, cellular growth pathway, proliferation and development pathway, cardiovascular signaling pathway, organismal growth and development pathway, neurotransmitter and nervous system signaling pathway, pathogen‐influenced signaling pathway, disease‐specific pathway, cellular stress and injury pathway, and cellular immune response and cancer pathway (Figure 1b, blue points). Figure 2 shows the most distinct signaling pathways between NAM and AM, including the S100 protein family signaling pathway, wound healing signaling pathway, phagosome formation pathway, stearate biosynthesis I pathway, focal adhesion kinase (FAK) signaling pathway, epithelial mesenchymal transition (EMT) regulation signaling pathway, cAMP‐response element‐binding protein (CREB) signaling pathway, tumor microenvironment signaling pathway, and natural killer cell signaling pathway.

**TABLE 1:** Clinical characteristics of patients undergoing transcriptome sequencing.

Variables	AM (*n*/%)	NAM (*n*/%)
Patients	15/100	8/100
Sex		
Male	7/46.7	4/50.0
Female	8/53.3	4/50.0
Age at diagnosis (years)		
<65	7/46.7	5/62.5
≥65	8/53.3	3/37.5
T category (Breslow thickness, mm)		
T1 (≤1.0)	2/13.3	2/25.0
T2 (1.1–2.0)	3/20.0	0/0.0
T3 (2.1–4.0)	3/20.0	2/25.0
T4 (>4.0)	7/46.7	4/50.0
Lymph node metastasis		
Negative	10/66.7	6/75.0
Positive	5/33.3	2/25.0
Ulceration		
Negative	5/33.3	5/62.5
Positive	10/66.7	3/37.5
Lymphovascular invasion		
Negative	14/93.3	8/100.0
Positive	1/6.7	0/0.0
Staging		
0	0/0.0	1/12.5
I	4/26.7	2/25.0
II	5/33.3	2/25.0
III	4/26.7	2/25.0
IV	2/13.3	1/12.5

Abbreviations: AM, acral melanoma; NAM, nonacral melanoma.

**FIGURE 3:**
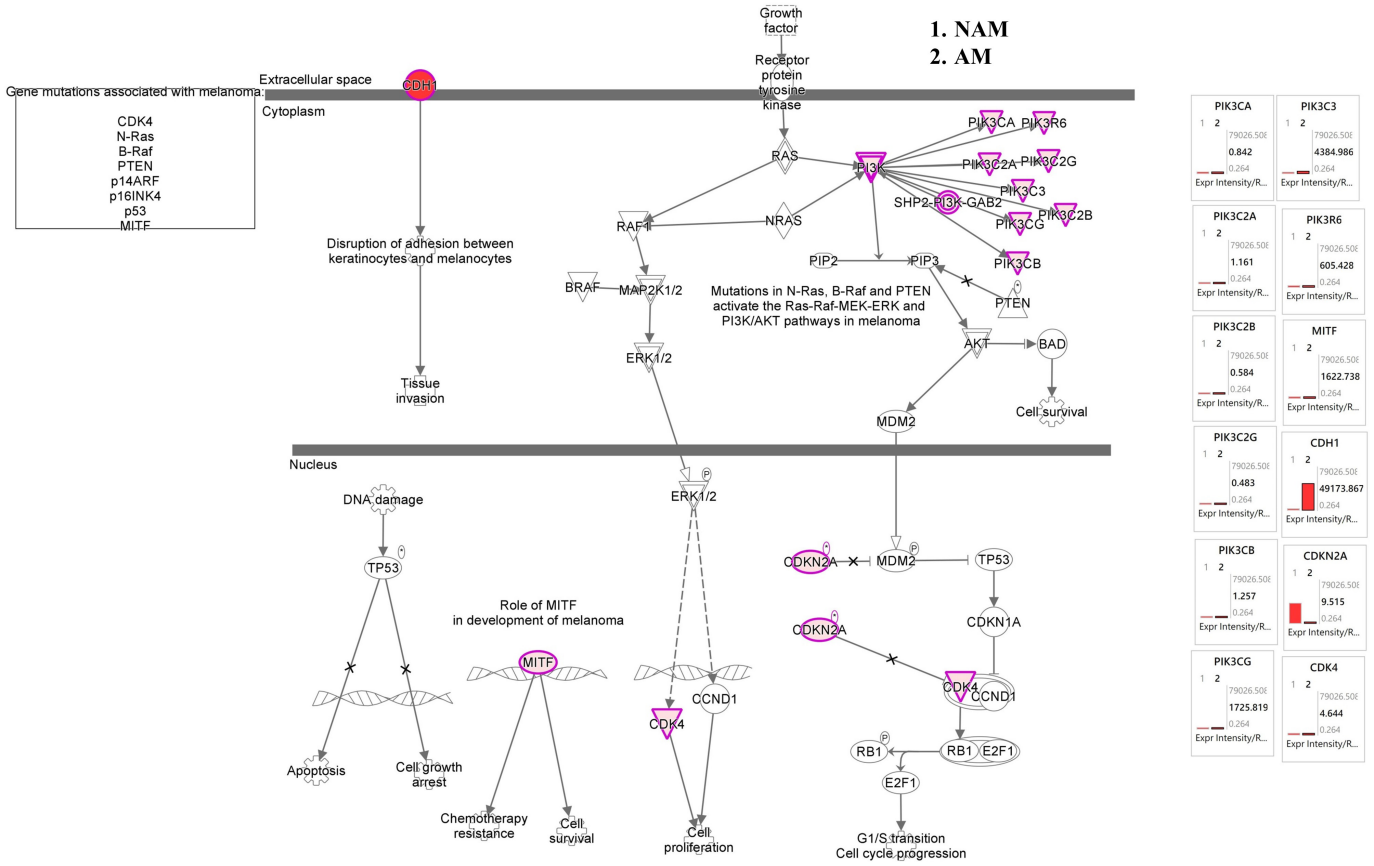
Melanoma and cell cycle mechanistic networks with multiple affected cell cycle signaling networks, predicting the activated state of the network on the basis of transcriptome data, with subsequent predicted effects on downstream effector molecules. The figure shows the differences in cell cycle family networks between acral melanoma (AM) and nonacral melanoma (NAM). In NAM, the expression of genes CDK2, CDK5, CDK5RAP2, CDK11A, CDK11B, CDK20, CDKN2A, and E2F3 was significantly upregulated. In AM, the expression of genes CDK7, CDK8P2, CDK16, CDKAL1, CDH1, E2F6, E2F8, and MITF was significantly upregulated.

**FIGURE 4:**
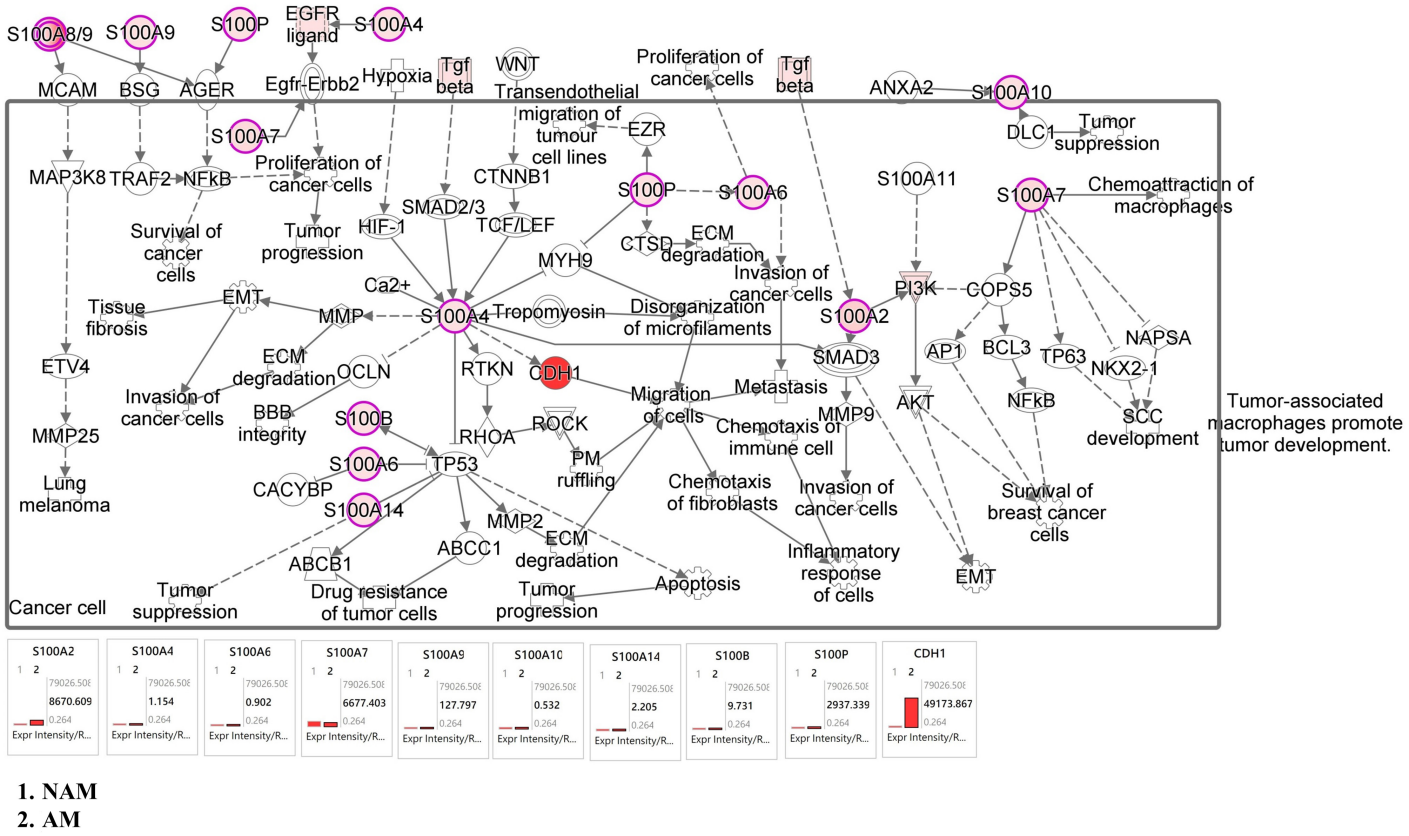
S100 mechanistic networks with multiple affected S100 signaling networks, predicting the activated state of the network on the basis of transcriptome data, with subsequent predicted effects on downstream effector molecules. In NAM, the expression of genes S100A1, S100A4, S100A7, S100A13, and S100PBP was significantly upregulated. In AM, the expression of genes S100A2, S100A7A, S100A8, and S100P was significantly upregulated. However, the expression of gene S100B did not differ between nonacral melanoma (NAM) and acral melanoma (AM).

In this study, we examined the differences in the melanoma signaling pathway, cell cycle signaling pathway, S100 protein family signaling pathway, cytokine and immune modulator signaling pathway, and PD‐1/PD‐L1 signaling pathway between AM and NAM. Figure 3 depicts our NGS analysis results for the cell cycle signaling pathway and melanoma signaling pathway. The results indicate that the expression of the genes of CDK2, CDK5, CDK5RAP2, CDK11A, CDK11B, CDK20, CDKN2A, and E2F3 was significantly upregulated in NAM and the expression of the genes of CDK7, CDK8P2, CDK16, CDKAL1, CDH1, E2F6, E2F8, and MITF was significantly upregulated in AM. As shown in Figure 4, the expression of the genes of S100A1, S100A4, S100A7, S100A13, and S100PBP was significantly upregulated in NAM and the expression of the genes of S100A2, S100A7A, S100A8, and S100P was significantly upregulated in AM. However, the expression of the gene of S100B did not significantly differ between NAM and AM.

**FIGURE 5:**
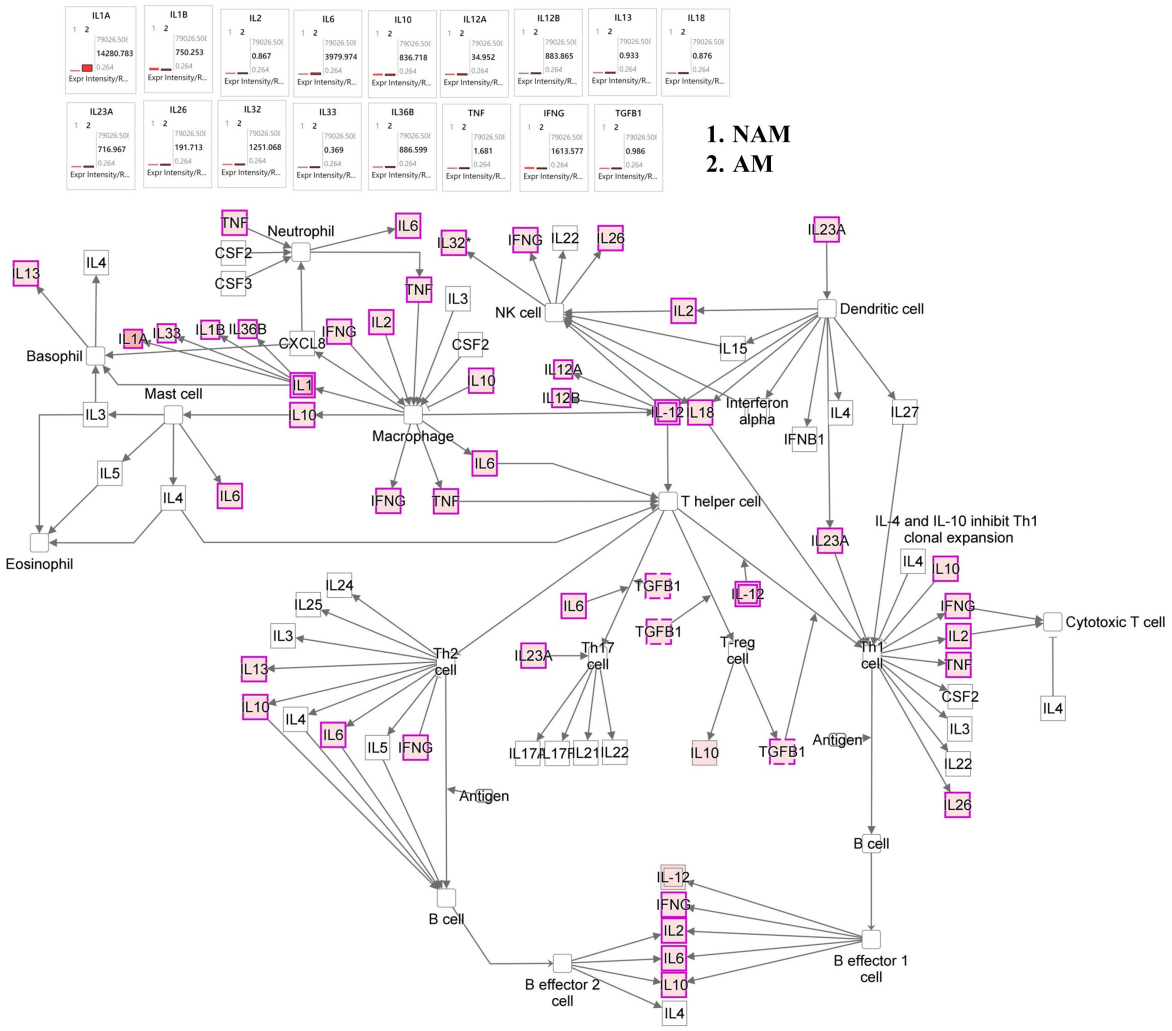
Immune modulator mechanistic networks with multiple affected melanoma signaling networks, predicting the activated state of the network on the basis of transcriptome data, with subsequent predicted effects on downstream effector molecules. The figure shows the differences in melanoma networks between acral melanoma (AM) and nonacral melanoma (NAM). In NAM, the expression of genes IL1B, IL2, IL10, IL12A, IL13, IL26, IL36, IFNG, and TGFB1 was significantly upregulated. In AM, the expression of genes IL1A, IL6, and IL32 was significantly upregulated. According to the results of human leukocyte antigen (HLA) gene analysis, the expression of genes HLA‐A, HLA‐B, HLA‐DMA, HLA‐DMB, and HLA‐K was significantly upregulated in NAM.

**FIGURE 6:**
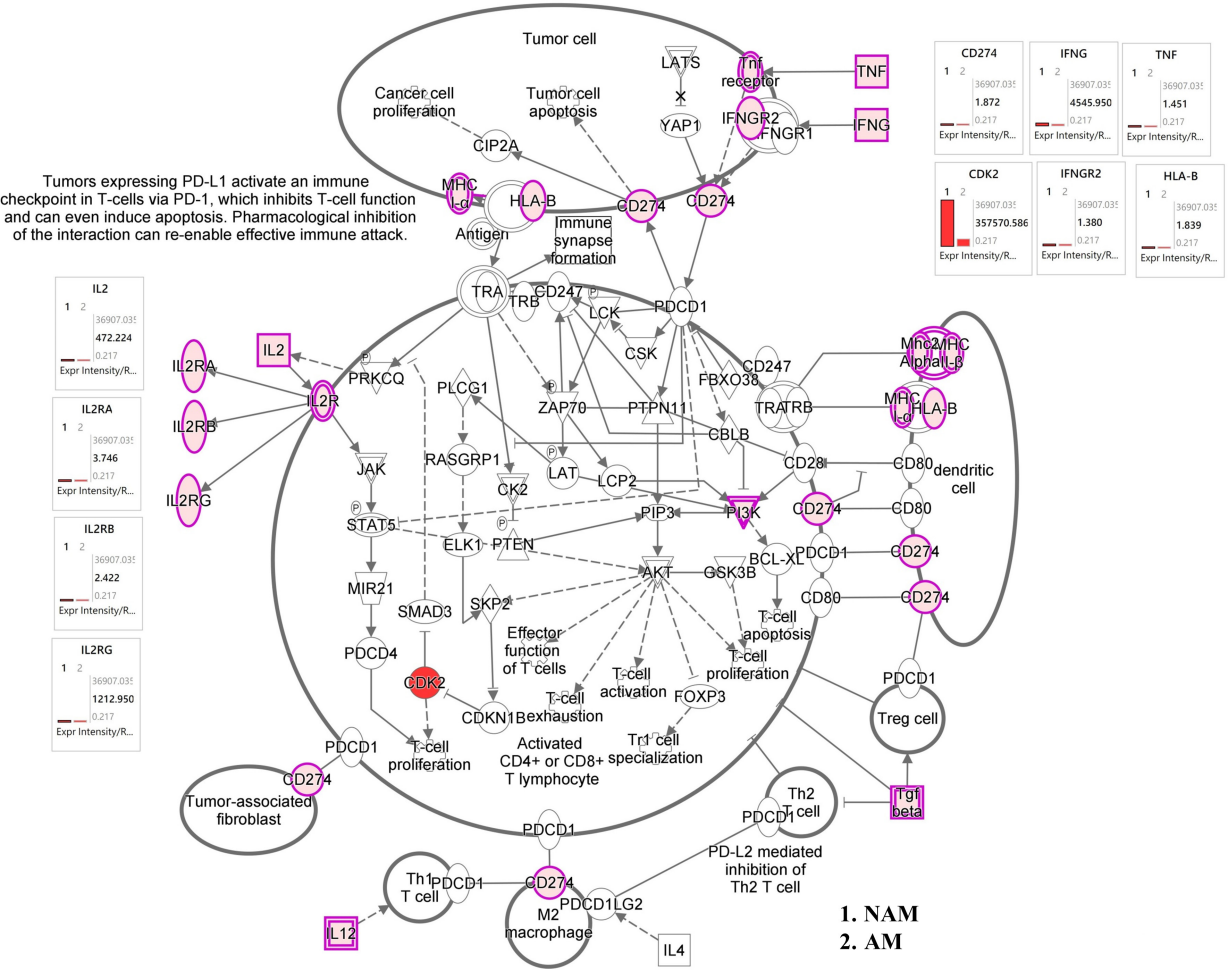
Programmed cell death protein 1/programmed cell death 1 ligand 1 (PD‐1/PD‐L1) mechanistic networks with multiple affected immune modulator signaling networks, predicting the activated state of the network on the basis of transcriptome data, with subsequent predicted effects on downstream effector molecules. The figure shows the differences in immune modulator networks between acral melanoma (AM) and nonacral melanoma (NAM). According to the results of PD‐1/PD‐L1 signaling analysis, the expression of genes IL2RA, IL2RB, IL2RG, HLA‐A, HLA‐B, CD274, and CDK2 was significantly upregulated in NAM.

Figure 5 presents the results of our immune modulator signaling analysis. According to these results, the expression of the genes of IL1B, IL2, IL10, IL12A, IL13, IL26, IL36, IFNG, and TGFB1 was significantly upregulated in NAM and the expression of the genes of IL1A, IL6, and IL32 was significantly upregulated in AM. According to the results of our human leukocyte antigen (HLA) gene analysis, the expression of the genes of HLA‐A, HLA‐B, HLA‐DMA, HLA‐DMB, and HLA‐K was significantly upregulated in NAM. Similarly, according to the results of our PD‐1/PD‐L1 signaling analysis (Figure 6), the expression of the genes of IL2RA, IL2RB, IL2RG, HLA‐A, HLA‐B, CD274, and CDK2 was significantly upregulated in NAM. Figure S1 shows the heat maps of the genes involved in S100 protein family signaling, cell cycle and melanoma signaling, and immune modulator signaling pathway. Table S1 presents the raw sequencing data.

### 3.2 Validation of immunogenicity differences between NAM and AM: Insights from immunohistochemistry and qPCR analyses

**FIGURE 7:**
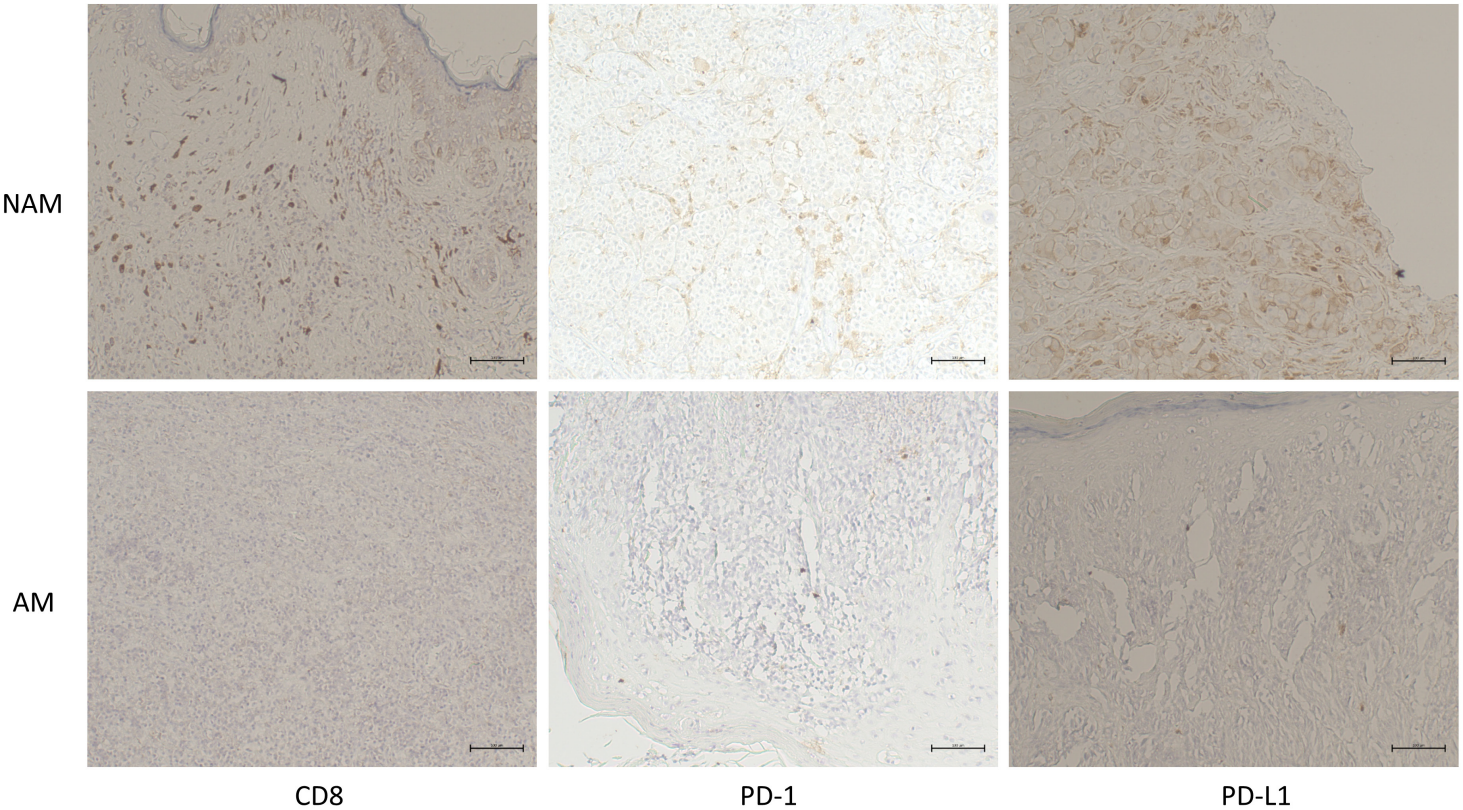
Immunohistochemistry analyses of CD8, programmed cell death protein 1 (PD‐1), programmed cell death 1 ligand 1 (PD‐L1) on both nonacral melanoma (NAM) and acral melanoma (AM) tumor samples. The results from representative cases are shown to reveal a higher expression of CD8, PD‐1, and PD‐L1 (brown color) in the NAM samples compared with the AM samples. Bars = 100 μm.

**FIGURE 8:**
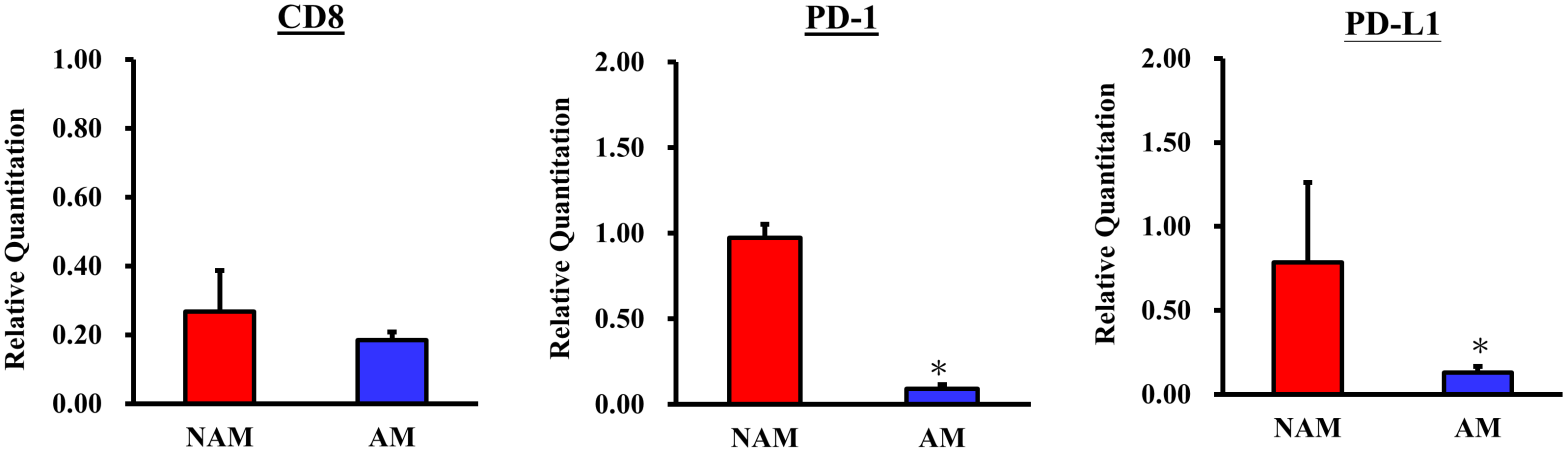
Quantitative polymerase chain reaction analyses targeting CD8, programmed cell death protein 1 (PD‐1), and programmed cell death 1 ligand 1 (PD‐L1) expression of both nonacral melanoma (NAM) and acral melanoma (AM) tumor samples. The findings revealed higher expression of these CD8, PD‐1, and PD‐L1 immune markers in the NAM samples compared with the AM samples. *p‐value <0.05 was considered to be statistically significant.

Subsequently, we undertook immunohistochemistry analyses of CD8, PD‐1, and PD‐L1 on both NAM and AM samples. In Figure 7, the results from representative cases revealed higher expression of CD8, PD‐1, and PD‐L1 in the NAM samples compared with the AM samples. We also performed qPCR analyses targeting CD8, PD‐1, and PD‐L1. In Figure 8, the qPCR findings align with our immunohistochemistry results, revealing an increased expression of these markers in NAM samples. This finding further corroborates our initial assertion that the immune response in AM samples is comparatively weaker. Together, these results provide substantial evidence to support the hypothesis that NAM exhibits a more robust antimelanoma immune response than AM.

## 4 DISCUSSION

Despite global advancements in melanoma treatment, researchers must recognize the unique challenges posed by regional and ethnic variations in disease presentation, particularly in Asian countries such as Taiwan; these observations indicate the necessity of a more geographically and ethnically tailored approach.^27^ Continued investment in research and healthcare infrastructure is essential to enhance the outcomes of melanoma. In our previous WES study, we discovered a large number of structural changes in Asian patients with melanoma, and low UV signatures were observed in both AM and NAM. We also observed significant differences in signatures 7, 21, and 22 as well as in TMB and MSI.^15^ In this study, we used transcriptome sequencing to further examine the disparities in RNA expression between AM and NAM.

Our transcriptome analysis results confirmed disparities in gene count and regulatory pathways between NAM and AM. These disparities may be attributable to DNA segment mutations that alter the expression levels of RNA. Through IPA analysis, we identified the following pathways as the most significantly different regulatory pathways between NAM and AM: S100 protein family signaling pathway, wound healing signaling pathway, phagosome formation pathway, stearate biosynthesis I pathway, FAK signaling pathway, EMT regulation signaling pathway, CREB signaling pathway, tumor microenvironment signaling pathway, natural killer cell signaling pathway, aldosterone signaling pathway, and interleukin 8 (IL‐8) signaling pathway.

The S100 protein family is valuable for staging, prognostic assessment, treatment efficacy evaluation, and recurrence prediction in malignant melanoma.^28^ Harpio et al.^29^ emphasized the pivotal role of the S100 protein family, particularly S100B, in therapeutic strategies. Nevertheless, research of the gene expression of the S100 protein family in NAM and AM remains limited. In the present study, as shown in Figure 4, the expression of the genes of S100A1, S100A4, S100A7, S100A13, and S100PBP was significantly upregulated in NAM, and the expression of the genes of S100A2, S100A7A, S100A8, and S100P was significantly upregulated in AM. These results indicate a correlation between the S100 protein family and melanoma genesis, with distinct gene expression patterns observed in NAM and AM. Notably, the expression of the gene of S100B did not significantly differ between NAM and AM.

Many studies have indicated that NAM results from the accumulation of mutations induced by exposure to UV radiation, whereas AM lacks these mutations and exhibits a range of structural variations.^16^, ^19^ Forschner et al.^30^ reported the amplification of MYC, TERT, CCND3, RICTOR, and CDK4 and the deletion of CDKN2A, CDKN2B, and PTEN in AM. Our findings are consistent with these results, confirming the upregulated expression of CDK4 in AM. This upregulation in CDK4 expression may be due to its amplification. Our data also indicate a significant increase in the gene expression of CDKN2A in NAM, with the opposite trend observed in AM. This reduced gene expression of CDKN2A in AM may be attributable to its deletion. These aforementioned findings suggest that AM is characterized by markedly elevated cell cycle aberrations.^4^, ^13^, ^19^ According to the International Cancer Genome Consortium/TCGA database, AM has a high abundance of structural variations in CCND1 and TERT. This is characterized by chromothripsis‐induced amplification of these genes, which is likely to lead to an increased expression of CCND1 and TERT.^31^, ^32^ By contrast, our study revealed no significant difference in the gene expression of CCND1 and TERT between AM and NAM. This discrepancy suggests a racial difference between Taiwanese and Western populations. CDK4/6 inhibitors, which have been employed in clinical practice, have demonstrated promising preliminary evidence of activity in the treatment of melanoma. We have systematically collated data from clinicaltrials.gov concerning trials involving CDK4/6 inhibitors in melanoma. The supplementary Table S2 delineates completed clinical studies and the supplementary Table S3 elucidates ongoing clinical trials.

In recent years, advancements in immunotherapy have led to major improvements in the prognosis of patients with melanoma. Nevertheless, multiple reports have indicated a low therapeutic response in patients with AM. Studies examining differences in immunogenicity between NAM and AM in Asian populations are sparse. In a study conducted in South Korea, Kim et al.^33^ reported that the plasma cytokine levels of both anti‐inflammatory cytokines (IL‐4, IL‐5, IL‐10, and IL‐13) and inflammatory cytokines (IL‐2, IL‐12, IFN‐γ, and TNF‐α) were higher in NAM than in AM. In this study, the results indicated that the expression of the genes of IL1B, ILDR1, IL1RAPL1, IL2, IL2RG, IL3RA, IL4R, IL6STP1, IL7, IL10, IL12A, IL21R‐AS1, IL36B, IFNG, TNFRSF6B, TNFRSF9, TNFRSF10B, TNFRSF11B, TNFRSF18, CD274, and CDK2 was significantly upregulated in NAM, whereas the expression of the genes of IL1A, IL6, IL6ST, IL11, IL18, IL23R, IL31RA, and IL32 was significantly upregulated in AM (Figures 5 and 6). According to the results of our HLA gene analysis, the expression of the genes of HLA‐A, HLA‐B, HLA‐DMA, HLA‐DMB, and HLA‐K was significantly upregulated in NAM. Overall, our tumor tissue results are largely consistent with the findings of Kim et al.,^33^ who reported elevated levels of IL‐2, IL‐12A, and IFNG in the plasma samples of patients with NAM.

In this study, the results revealed a significant increase in the expression of the genes of CDH1 and MITF in AM, which strongly correlates with poor immunity in patients with melanoma.^34^, ^35^, ^36^ In NAM, the expression of the genes of IL‐2 and IL‐2R significantly increases, which may result in increased T‐cell activity and enhanced PD‐1 mAb levels.^37^, ^38^ An increase in the expression of CDK2 leads to the proliferation of T cells.^39^, ^40^ In this study, we discovered a significant increase in the expression of the gene of CD274 on chromosome 9 in tumor cells as well as an increase in the expression of the gene of IFNG, thereby contributing to the expression and upregulation of PD‐L1 messenger RNA.^41^, ^42^ These findings indicate the enhanced activity of T cells. Our results also indicate that NAM was associated with a significant increase in the expression of the genes of HLA‐A, HLA‐B, HLA‐DMA, HLA‐DMB, and HLA‐K. These findings are consistent with those of previous Taiwanese studies, suggesting enhanced immunogenicity in NAM^43^ and reduced immunogenicity in AM.^43^, ^44^ Our findings are also consistent with those of our previous WES study, which reported an increase in mutational signatures 7 and 21 as well as in TMB and MSI in NAM. According to previous studies, an increase in mutational signatures 7 and 21, along with an increase in TMB and MSI, is associated with improved responses to immunotherapy.

In this study, we comprehensively investigated genetic variations between NAM and AM. Our findings revealed distinct expression profiles for the two subtypes. Specifically, we observed increases in CDK4 expression and cell cycle variability in AM and an increase in immunogenicity in NAM. These novel insights contribute to our understanding of the pathogenesis of different melanoma subtypes and can serve as a valuable reference for future research.

## CONFLICT OF INTEREST STATEMENT

None declared.

## Supporting information


Supplementary Figure S1.

Supplementary Table S1.

Supplementary Table S2.

Supplementary Table S3.

Figure S1 Caption.
