# Supplementary figures and images for: Comparative transcriptomic analysis reveals differences in gene expression and regulatory pathways between nonacral and acral melanoma in Asian individuals
Source: J Dermatol. 2024 Mar 12;51(5):659–70. doi: 10.1111/1346-8138.17187 (PMC11484150; doi:10.1111/1346-8138.17187)

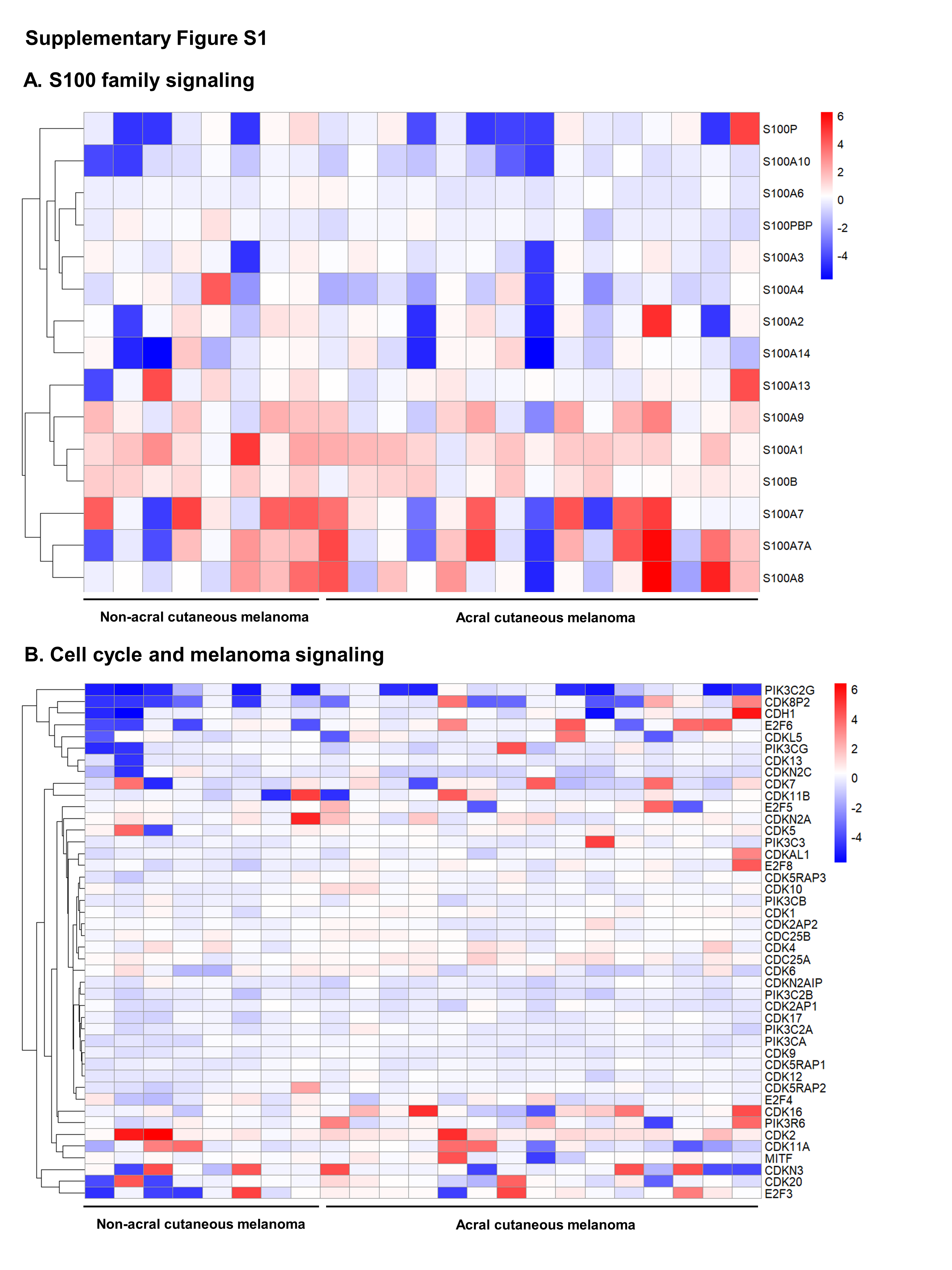

Supplement: Supplementary file 1 — Supplementary Figure S1. [file JDE-51--s004.zip › Supplementary Figure S1-1.TIF]

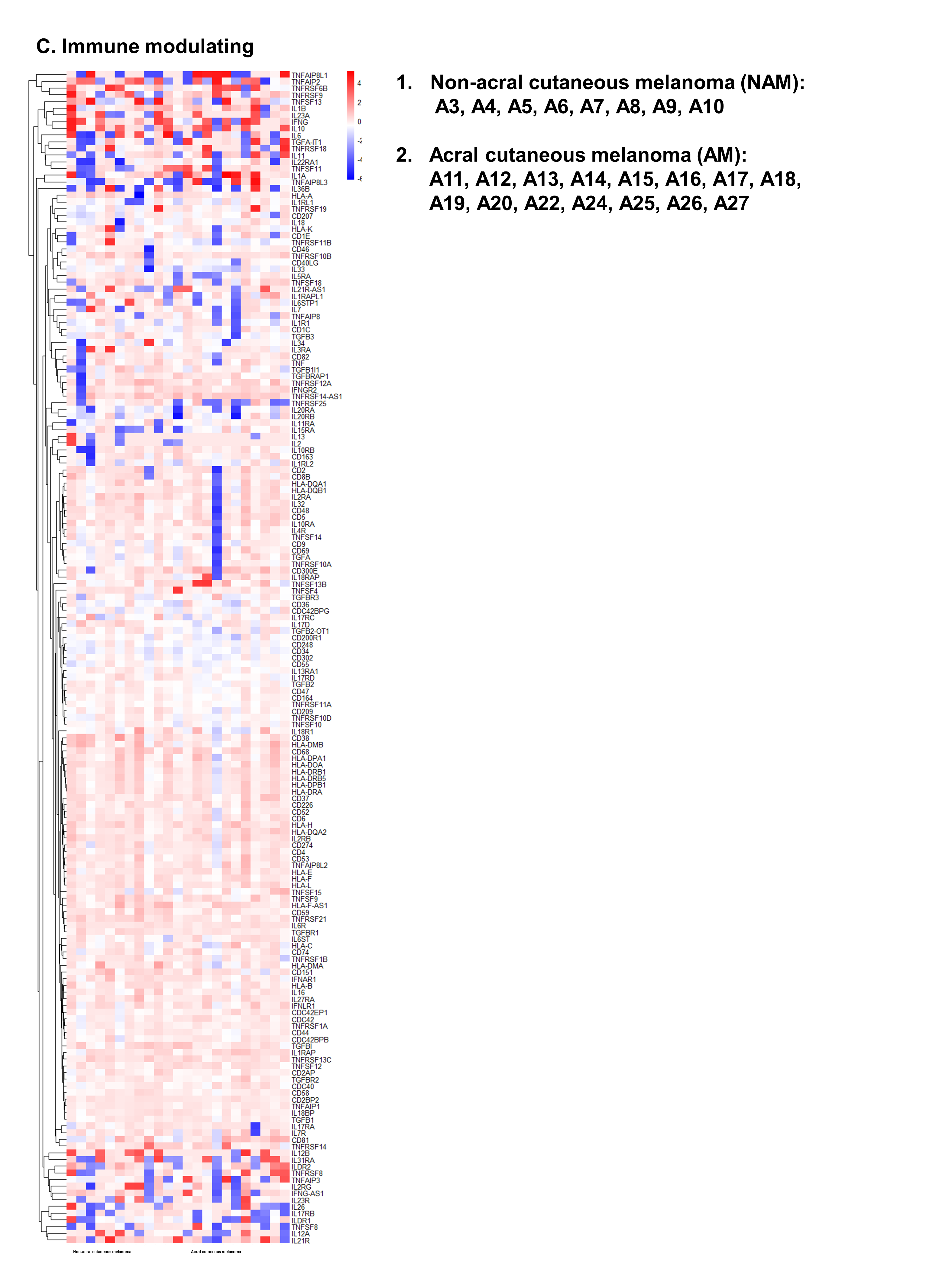

Supplement: Supplementary file 1 — Supplementary Figure S1. [file JDE-51--s004.zip › Supplementary Figure S1-2.TIF]
