# Supplementary material for: Comparative transcriptomic analysis reveals differences in gene expression and regulatory pathways between nonacral and acral melanoma in Asian individuals
Source: J Dermatol. 2024 Mar 12;51(5):659–70. doi: 10.1111/1346-8138.17187 (PMC11484150; doi:10.1111/1346-8138.17187)
Supplement: Supplementary file 3 — Supplementary Table S2. [file JDE-51--s002.docx]

**Supplementary Table S2**

Completed clinical trials with CDK4/6 inhibitors in melanoma

| Investigated treatment | Phase | Location | Setting | Patient number | Results | Study |
| --- | --- | --- | --- | --- | --- | --- |
| Palbociclib | II | China | Advanced AM with CDK4 or/and CCND1 gain or/and CDKN2A loss | 15 | Preliminary efficacy and an acceptable safety profile. | NCT03454919^1^ |
| Palbociclib/  Vemurafenib | I/II | France | Metastatic melanoma with BRAF^V600 mutant^ and CDKN2A Loss and Expression of Rb | 18 | A significant clinical benefit was achieved in pretreated patients. | NCT02202200  (OPTIMUM)^2^ |
| Abemaciclib | II | United States | Melanoma with brain metastasis | 162 | Pending formal reports | NCT02308020 |
| Abemaciclib /LY3214996 | I | United States, Australia, France, Japan | Metastatic NRAS or BRAF mutant melanoma | 210 | Pending formal reports | NCT02857270 |
| Ribociclib/  Binimetinib | I/II | United States, Australia, Europe | Advanced or metastatic NRAS mutant melanoma | 102 | Safely administered and clinically active in patients with NRAS-mutant melanoma. | NCT01781572^3^ |
| Ribociclib/ Binimetinib | I/II | United States, Australia, Europe, Korea, Japan | NRAS mutant melanoma | 14 | Promising preliminary antitumor activity in patients with NRAS mutant melanoma | NCT01719380^4^ |
| Ribociclib/ Encorafenib | I/II | United States, Australia, Canada, Netherlands | Metastatic BRAF mutant melanoma | 28 | Pending formal reports | NCT01777776 |
| Ribociclib / Encorafenib/  Binimetinib | II | United States, Australia, Canada, Europe | Metastatic or unresectable BRAF^V600 mutant^ melanoma | 160 | Pending formal reports | NCT02159066  (LOGIC-2) |

**References**

1 Mao L, Dai J, Cao Y et al. Palbociclib in advanced acral melanoma with genetic aberrations in the cyclin-dependent kinase 4 pathway. *European Journal of Cancer* 2021; **148**: 297-306.

2 Louveau B, Resche-Rigon M, Lesimple T et al. Phase I–II Open-Label Multicenter Study of Palbociclib+ Vemurafenib in BRAF V600MUT Metastatic Melanoma Patients: Uncovering CHEK2 as a Major Response Mechanism. *Clinical Cancer Research* 2021; **27**(14): 3876-3883.

3 Schuler M, Zimmer L, Kim KB et al. Phase Ib/II trial of ribociclib in combination with binimetinib in patients with NRAS-mutant melanoma. *Clinical Cancer Research* 2022; **28**(14): 3002-3010.

4 Sosman JA, Kittaneh M, Lolkema MP et al. A phase 1b/2 study of LEE011 in combination with binimetinib (MEK162) in patients with NRAS-mutant melanoma: early encouraging clinical activity. American Society of Clinical Oncology; 2014.
