# Supplementary material for: Comparative transcriptomic analysis reveals differences in gene expression and regulatory pathways between nonacral and acral melanoma in Asian individuals
Source: J Dermatol. 2024 Mar 12;51(5):659–70. doi: 10.1111/1346-8138.17187 (PMC11484150; doi:10.1111/1346-8138.17187)
Supplement: Supplementary file 4 — Supplementary Table S3. [file JDE-51--s005.docx]

**Supplementary Table S3**

Ongoing clinical trial with CDK4/6 inhibitors in melanoma. DLTs, Dose-limiting toxicities; ORR, objective response rate

| Investigated treatment | Phase | Location | Setting | Patient number | Primary Outcome | Study | Current Status | Estimated End |
| --- | --- | --- | --- | --- | --- | --- | --- | --- |
| Palbociclib /Encorafenib /Binimetinib | I/II | Australia | Metastatic BRAF mutant melanoma | 78 | DLTs | NCT04720768 (CELEBRATE) | Recruiting | 2024-12 |
| Palbociclib / Ulixertinib​ | I | United States | Metastatic RAS mutant and NF1 mutant melanoma | 45  (non-melanoma specific) | DLTs | NCT03454035 | Recruiting | 2026-04 |
| Palbociclib | II | United States | Metastatic or recurrent melanoma | 6452  (non-melanoma specific) | ORR | NCT02465060  (MATCH) | Active, not recruiting | 2025-12 |
| Abemaciclib / LY3300054 | I | United States, Canada, Europe, Korea and Taiwan | Metastatic melanoma | 215  (non-melanoma specific) | DLTs | NCT02791334  (PACT) | Active, not recruiting | 2024-03 |
| Ribociclib / LXH254 | II | United States, Argentina, Australia, Europe | Unresectable or metastatic BRAF^V600^ or NRAS mutant melanoma | 134 | ORR | NCT04417621 | Active, not recruiting | 2024-04 |
| Ribociclib /Trametinib | II | Australia | Metastatic melanoma | 1000 | ORR | NCT02645149  (MatchMel)^1^ | Recruiting | 2028-12 |
| Ribociclib/ LXH254 | I | United States, Australia, Europe, Korea | Metastatic NRAS mutant melanoma | 241  (non-melanoma specific) | DLTs and safety | NCT02974725 | Active, not recruiting | 2024-04 |

**References**

1 Nassar KW, Hintzsche JD, Bagby SM et al. Targeting CDK4/6 Represents a Therapeutic Vulnerability in Acquired BRAF/MEK Inhibitor–Resistant Melanoma. *Molecular cancer therapeutics* 2021; **20**(10): 2049-2060.
