# Supplementary material for: Comparative transcriptomic analysis reveals differences in gene expression and regulatory pathways between nonacral and acral melanoma in Asian individuals
Source: J Dermatol. 2024 Mar 12;51(5):659–70. doi: 10.1111/1346-8138.17187 (PMC11484150; doi:10.1111/1346-8138.17187)
Supplement: Supplementary file 5 — Figure S1 Caption. [file JDE-51--s003.docx]

Figure S1. Heatmap of (A) S100 protein family genes, (B) cell cycle and melanoma signaling genes, and (C) immune modulator genes.
